# NMR Metabolite Profiles in Male Meat-Eaters, Fish-Eaters, Vegetarians and Vegans, and Comparison with MS Metabolite Profiles

**DOI:** 10.3390/metabo11020121

**Published:** 2021-02-20

**Authors:** Julie A. Schmidt, Georgina K. Fensom, Sabina Rinaldi, Augustin Scalbert, Marc J. Gunter, Michael V. Holmes, Timothy J. Key, Ruth C. Travis

**Affiliations:** 1 Cancer Epidemiology Unit, Nuffield Department of Population Health, University of Oxford, Oxford OX3 7LF, UK; georgina.fensom@qeh.ox.ac.uk (G.K.F.); tim.key@ndph.ox.ac.uk (T.J.K.); ruth.travis@ndph.ox.ac.uk (R.C.T.); 2 Department of International Development, University of Oxford, Oxford OX1 3TB, UK; 3 Section of Nutrition and Metabolism, International Agency for Research on Cancer, 69372 Lyon, France; rinaldis@iarc.fr (S.R.); scalberta@iarc.fr (A.S.); gunterm@iarc.fr (M.J.G.); 4 Medical Research Council Population Health Research Unit, Nuffield Department of Population Health, University of Oxford, Oxford OX3 7LF, UK; michael.holmes@ndph.ox.ac.uk; 5 Clinical Trial Service Unit & Epidemiological Studies Unit, Nuffield Department of Population Health, University of Oxford, Oxford OX3 7LF, UK

**Keywords:** European Prospective Investigation into Cancer and Nutrition (EPIC)-Oxford, fish-eaters, mass spectrometry (MS), meat-eaters, metabolomics, nuclear magnetic resonance (NMR), vegans, vegetarians

## Abstract

Metabolomics may help to elucidate mechanisms underlying diet-disease relationships and identify novel risk factors for disease. To inform the design and interpretation of such research, evidence on diet-metabolite associations and cross-assay comparisons is needed. We aimed to compare nuclear magnetic resonance (NMR) metabolite profiles between meat-eaters, fish-eaters, vegetarians and vegans, and to compare NMR measurements to those from mass spectrometry (MS), clinical chemistry and capillary gas-liquid chromatography (GC). We quantified 207 serum NMR metabolite measures in 286 male participants of the European Prospective Investigation into Cancer and Nutrition (EPIC)-Oxford cohort. Using univariate and multivariate analyses, we found that metabolite profiles varied by diet group, especially for vegans; the main differences compared to meat-eaters were lower levels of docosahexaenoic acid, total n-3 and saturated fatty acids, cholesterol and triglycerides in very-low-density lipoproteins, various lipid factions in high-density lipoprotein, sphingomyelins, tyrosine and creatinine, and higher levels of linoleic acid, total n-6, polyunsaturated fatty acids and alanine. Levels in fish-eaters and vegetarians differed by metabolite measure. Concentrations of 13 metabolites measured using both NMR and MS, clinical chemistry or GC were mostly similar. In summary, vegans’ metabolite profiles were markedly different to those of men consuming animal products. The studied metabolomics platforms are complementary, with limited overlap between metabolite classes.

## 1. Introduction

Diets with reduced intakes of animal products, including vegetarian and vegan diets, are becoming increasingly popular in the Western world for ethical, environmental and health reasons [1]. Prospective epidemiological evidence suggests that individuals excluding some (e.g., fish-eaters and vegetarian) or all animal products (vegans) from their diets may have different risks of some major non-communicable diseases compared to meat-eaters. This includes lower risks of coronary heart disease [2,3,4], some cancers [5,6,7,8], and diabetes [9,10] but possibly an increased risk of stroke [4] and fractures [11], in some or all of the non-meat-eating diet groups. Metabolomics has the potential to reveal underlying mechanisms in such diet-disease relationships [12], because metabolite concentrations reflect dietary, lifestyle, environmental and genetic factors as well as disease states [13]. Indeed, associations between metabolite concentrations and risk of diseases, e.g., cardiovascular events [14,15,16], diabetes [17] and some cancers [18,19], have been reported. A better understanding of how metabolite profiles differ between diet groups (defined based on the type of animal products consumed) will support mechanistic research on diet and disease risk. Previous studies, including research conducted within the Oxford arm of the European Prospective Investigation into Cancer and Nutrition (EPIC-Oxford), have found that the blood metabolite profile of individuals not habitually consuming meat significantly differs from the profile of those who consume meat [20,21,22,23,24]. We observed that the plasma metabolite profile, assessed using a targeted mass spectrometry (MS) assay (Absolute*IDQ*^®^ p180 Kit, Biocrates Life Sciences AG, Innsbruck, Austria; 118 metabolites, 379 men), differed between meat-eaters, fish-eaters, vegetarians and especially vegans, primarily due to lower concentrations of glycerophospholipids and sphingomyelins in vegans [20] as well as differences in concentrations of acylcarnitines and amino acids between diet groups [20,21]. Three smaller studies (*n* = 120, 36 and 31 individuals), which investigated circulating metabolite profiles of habitual diet groups, using nuclear magnetic resonance profiles (NMR; 800 MHz using a Bruker Avance III HD spectrometer; ~70 metabolites) or MS (liquid- and/or gas-chromatography coupled with MS; 66 metabolites), have reported differences between diet groups in amino acids, fatty acids, creatine, trimethylamine and xenobiotics [22,23,24]. Similarly, differences in the urine metabolome between individuals from different diet groups have been reported [25,26].

Here, we aimed to compare serum metabolite profiles between male meat-eaters, fish-eaters, vegetarians and vegans from EPIC-Oxford, using a high-throughput NMR platform (Nightingale Health Ltd., Helsinki, Finland), covering other parts of the blood metabolome than previous studies. To further aid planning and interpretation of studies of diet, metabolites and health outcomes, we additionally aimed to compare the measurements obtained using the NMR platform to those from MS (Biocrates Absolute*IDQ*^®^ p180 Kit) [20], clinical chemistry and capillary gas-liquid chromatography (GC) [27,28]. To our knowledge, this is the first study to characterize diet groups using the Nightingale platform and to compare metabolite levels measured using the Nightingale and Biocrates platforms. Both platforms are commonly used in prospective studies of metabolomics and disease risk [29], and the Nightingale platform measures a large number of metabolites which have not yet been studied in relation to diet groups, including lipoprotein subclass profiling with lipid concentrations within 14 subclasses.

## 2. Results

### 2.1. Participant and Sample Characteristics

This cross-sectional analysis included 286 men categorized as meat-eaters (who eat meat), fish-eaters (who eat fish but not meat), vegetarians (who eat neither meat nor fish) and vegans (who do not eat meat, fish, dairy or eggs, Figure S1). Participant characteristics and factors related to blood collection and handling by diet group are shown in Table 1. Meat-eaters had the highest body mass index (BMI) and waist circumference, and were most likely to be current smokers, while vegans were most likely to be very physically active and to drink less alcohol. Vegans had the lowest intakes of energy and saturated fatty acids (SFA), and the highest intakes of carbohydrate and polyunsaturated fatty acids (PUFA). Intake of dietary docosahexaenoic acid (DHA; C22:6 n-3, mainly from intake of fatty fish) was negligible in vegetarians and assumed to be zero in vegans. Most blood samples were taken before lunch time (1pm), and at blood collection meat-eaters had most recently had their last meal, followed by fish-eaters, vegetarians and vegans. Most blood samples from meat-eaters arrived at the laboratory by the end of the next working day; so did about half of the samples from vegetarians and vegans while the percentage was lowest in fish-eaters. 

We included 207 NMR metabolite measures in the analyses (Figure S1) and correlations between pairs of these metabolites are shown in Table S1.

**Figure 1:**
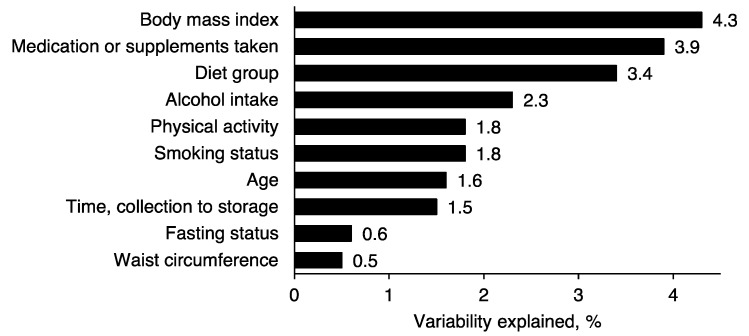
Variability in NMR metabolite data explained by diet group and other covariates. The percentage of the total variability explained by each variable was calculated using the Principal Component Partial R-square (PC-PR2) method [32], which accounts for all variables in the model.

The Principal Component Partial R-squared (PC-PR2) analysis [32] showed that together diet group, lifestyle and blood sampling and handling related variables explained 23.1% of the total variability in the NMR data. BMI explained the highest proportion (4.3%), after accounting for all other variables in the model. This was followed by use of medication or supplements on the day of blood collection (3.9%) and diet group (3.4%; Figure 1). Alcohol intake, physical activity, smoking status, age and time from blood collection to processing explained from 2.3 to 1.5%, while lower proportions were explained by fasting status (0.6%) and waist circumference (0.5%).

### 2.2. Metabolomics Profile by Diet Group—Univariate Analysis

Of the 207 NMR metabolite variables, 104 (50.2%) varied statistically significantly by diet group in the analysis of variance (ANOVA) after adjustment for confounding factors and correcting for multiple testing (Tables S2 and S3 shows the geometric mean metabolite measures on the original scale and standardised to the adjusted mean in meat-eaters, respectively). The metabolites that differed between diet groups included all lipid-related traits in the lipoprotein subclasses chylomicrons and extremely large (XXL) very-low-density lipoprotein (VLDL), large (L) VLDL and very large (XL) HDL (High-density lipoprotein), and all lipid-related traits except triglycerides for the subclass very small (XS) VLDL, intermediate-density lipoprotein (IDL) and L low-density lipoprotein (LDL). For all these, the lowest concentrations were observed in vegans and the highest were found in meat-eaters, except for XXL VLDL, and L VLDL, for which vegetarians had the highest concentrations. Moreover, all measurements differed significantly by diet group in the categories mean particle diameter, ratios of fatty acids to total fatty acids, and amino acids, except isoleucine and phenylalanine. Creatinine was highest in meat-eaters and lowest in vegans, with intermediate levels in fish-eaters and vegetarians.

**Figure 2:**
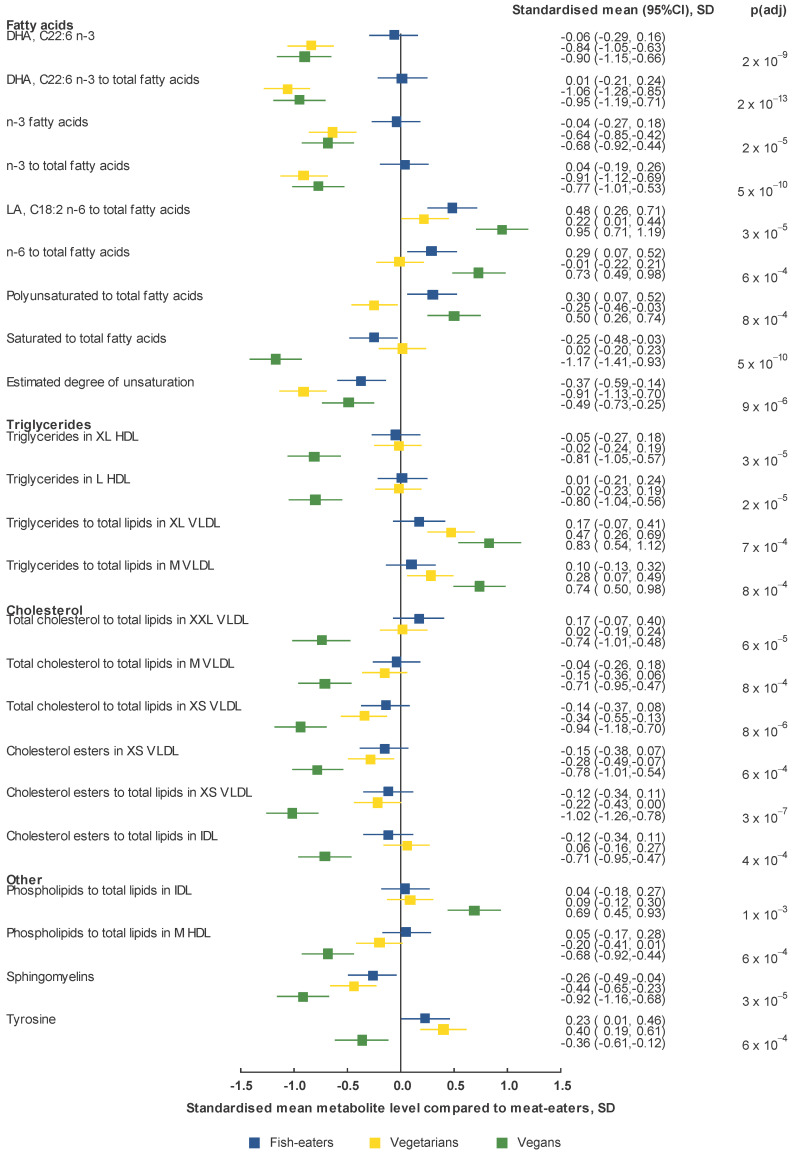
Geometric mean of selected NMR metabolite measures by diet group compared to meat-eaters. Means were standardized to the adjusted means in meat-eaters. *p*(*adj*) is the *p*-value after adjusting for multiple testing using a FDR of 0.05 on all 207 metabolite variables, and metabolites with *p*(*adj*) < 0.001 were included in the figure. All analyses were adjusted for age, BMI, smoking, alcohol, physical activity level, time since last meal at blood collection and time from blood collection to processing. Results for all NMR metabolite measures are shown in Table S3. Abbreviations: CI, confidence interval; DHA, docosahexaenoic acid; HDL, high-density lipoprotein; IDL, intermediate-density lipoprotein; L, large; LA, linoleic acid; M, medium; SD, standard deviation; VLDL, very-low-density lipoprotein; XL, extra-large; XS, extra-small; XXL, chylomicrons and extremely large.

Results for the 23 most significant associations (*p*-value after adjusting for multiple testing [*p*(*adj*)] < 0.001) are displayed in Figure 2 and Table 2.

Fatty acid concentrations and their ratios to total fatty acids showed some of the largest and most statistically significant differences between diet groups. For DHA and total n-3 fatty acids concentrations, and the corresponding ratios, meat-eaters and fish-eaters had the highest levels, with markedly lower levels in vegetarians and vegans. The ratios of linoleic acid (LA; C18:2 n-6) and total n-6 to total fatty acids were highest in vegans followed by fish-eaters, then vegetarians and meat-eaters. The ratio of SFA to total fatty acids was markedly lower in vegans than the other diet groups. The mean ratio in vegans was 1.17 (95% confidence interval 0.93, 1.41) standard deviations (SD) lower than that of meat-eaters, which was the most extreme difference observed in the study; the result for fish-eaters was −0.25 SD (−0.48, −0.03) and it was 0.02 SD (−0.20, 0.23) for vegetarians (Figure 2 and Figure S3). Furthermore, we observed the ratio of PUFA to total fatty acids and the degree of saturation among the top hits; both were lowest in vegetarians and highest in vegans and meat-eaters, respectively.

Triglyceride concentrations in various lipoprotein subtypes were lowest in vegans, with the strongest association observed in XL and L HDL for which we observed similar higher concentrations in meat-eaters, fish-eaters and vegetarians. For the ratio between triglycerides and total lipids, the difference by diet group depended on the lipoprotein subtype; vegans had the highest for all sizes of VLDL with consecutively lower values in vegetarians, fish-eaters and meat-eaters (except for XXL VLDL), while vegans had the lowest and vegetarians the largest ratios for XL HDL and L HDL.

Total cholesterol and its ratio to total lipids were mostly highest in meat-eaters and lowest in vegans. The largest difference was observed for the ratios of total cholesterol to total lipids in XXL VLDL, medium (M) VLDL and XS VLDL, for which ratios were highest in meat-eaters and fish-eaters, similar or slightly lower in vegetarians, and markedly lower in vegans. A similar pattern was seen for cholesterol esters and their ratio to total lipids, with the most pronounced differences by diet group being in cholesterol esters in XS VLDL, the ratios in XS VLDL and in IDL.

While the ratio of phospholipids to total fatty acids in IDL was higher in vegans and similarly lower in the other diet groups, the opposite was the case for the same ratio in M HDL. The concentration of sphingomyelins was highest in meat-eaters, followed by fish-eaters, then vegetarians and lowest in vegans. Among the amino acids, tyrosine showed the strongest variation by diet group; the concentrations were highest in vegetarians, followed by fish-eaters, then meat-eaters and lowest in vegans.

### 2.3. Metabolomics Profile by Diet Group—Multivariate Analysis

The multivariate analyses included 71 meat-eaters, 56 fish-eaters, 69 vegetarians and 41 vegans with complete NMR metabolite measurements.

**Figure 3:**
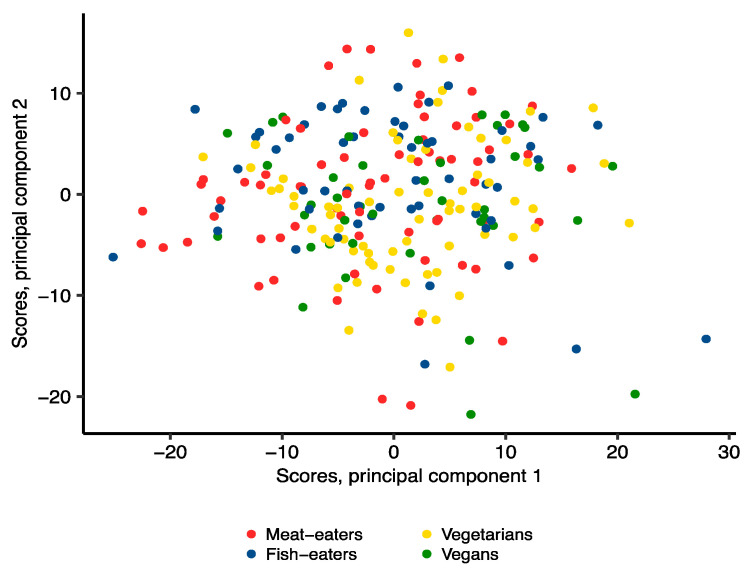
Score plot of principal component analysis of 207 NMR metabolite variables by diet group.

From the principal component analysis (PCA), the first two principal components explained 39.4% and 22.6% of the total variation in the NMR data, respectively. The score plots showed little separation between diet groups for the principal components (Figure 3). The loadings of each individual metabolite variable on the first two principal components are shown in Table S4.

**Figure 4:**
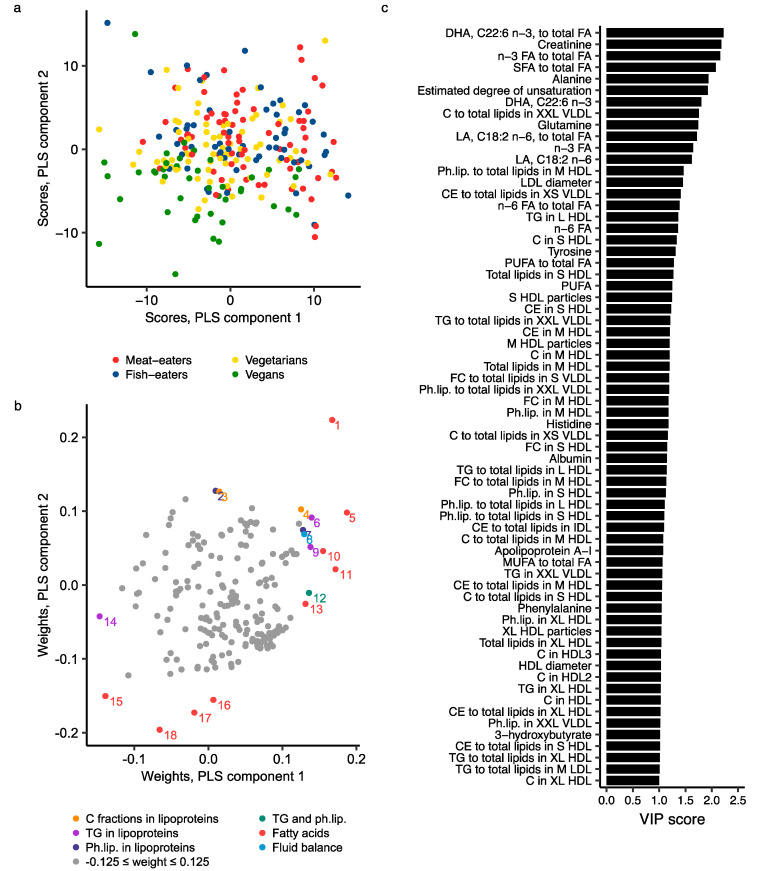
PLS model of 207 NMR metabolite measures by four diet groups. (**a**) Score plot of PLS components 1 and 2 coloured by diet group; (**b**) Weights plot of PLS components 1 and 2. Metabolites with weights more extreme than ±0.125 on either PLS component are coloured and labelled: 1, Saturated to total fatty acids; 2, Phospholipids to total lipids in S HDL; 3, Free cholesterol to total lipids in S HDL; 4, Cholesterol esters to total lipids in XS VLDL; 5, DHA, C22:6 n-3, to total fatty acids; 6, Triglycerides in L HDL; 7, Phospholipids to total lipids in M HDL; 8, Creatinine; 9, Triglycerides in XL HDL; 10, n-3 to total fatty acids; 11, DHA, C22:6 n-3; 12, Sphingomyelins; 13, n-3 fatty acids; 14, Triglycerides to total lipids in XL VLDL; 15, Linoleic acid, C18:2 n-6, to total fatty acids; 16, Polyunsaturated fatty acids; 17, n-6 fatty acids; 18, Linoleic acid, C18:2 n-6; (**c**) Variable influence on projection (VIP) scores, representing the contribution of metabolites to the PLS model; metabolites with VIP > 1 are included in the figure. Further details are given in Table S5. Abbreviations: C, total cholesterol; CE, cholesterol esters; C fractions, cholesterol fractions, i.e., total cholesterol, cholesterol esters and free cholesterols; DHA, docosahexaenoic acid; FA, fatty acids; FC, free cholesterol; HDL, high-density lipoprotein; IDL, intermediate-density lipoprotein; L, large; LA, linoleic acid; LDL, low-density lipoprotein; M, medium; MUFA, monounsaturated fatty acids; Ph.lip., phospholipids; PLS, partial least squares; PUFA, poly unsaturated fatty acids; S, small; SFA, saturated fatty acids; TG, triglycerides; VIP, variable influence on projection; VLDL, very-low-density lipoprotein; XL, extra-large; XS, extra-small; XXL, chylomicrons and extremely large.

The best partial least squares (PLS) regression model for the four diet groups contained seven components, explaining a total of 85.2% of the total variance in metabolite data, but it had low goodness of fit (R^2^Y = 0.323) and goodness of prediction (Q^2^Y = 0.185; Figure S2). No sign of overfitting was observed (Figure S3). The score plot of components 1 and 2 showed some separation of diet groups; meat- and fish-eaters had similar scores, while vegans separated somewhat from the other diet groups, with intermediate values in vegetarians (Figure 4a). The metabolite measures most important for differentiating the diet groups included fatty acids (absolute concentrations and/or ratios to total fatty acids of DHA [highest variable influence on projection (VIP) score of 2.2], total n-3, SFA, estimated degree of unsaturation, LA, total n-6 and PUFA), creatinine, amino acids (mainly alanine, glutamine and tyrosine), components of HDL subclasses (mainly small (S) and M HDL) and VLDL subclasses, LDL diameter, and sphingomyelins, albumin and apolipoprotein A-I concentrations (Figure 4b,c; Table S5).

**Figure 5:**
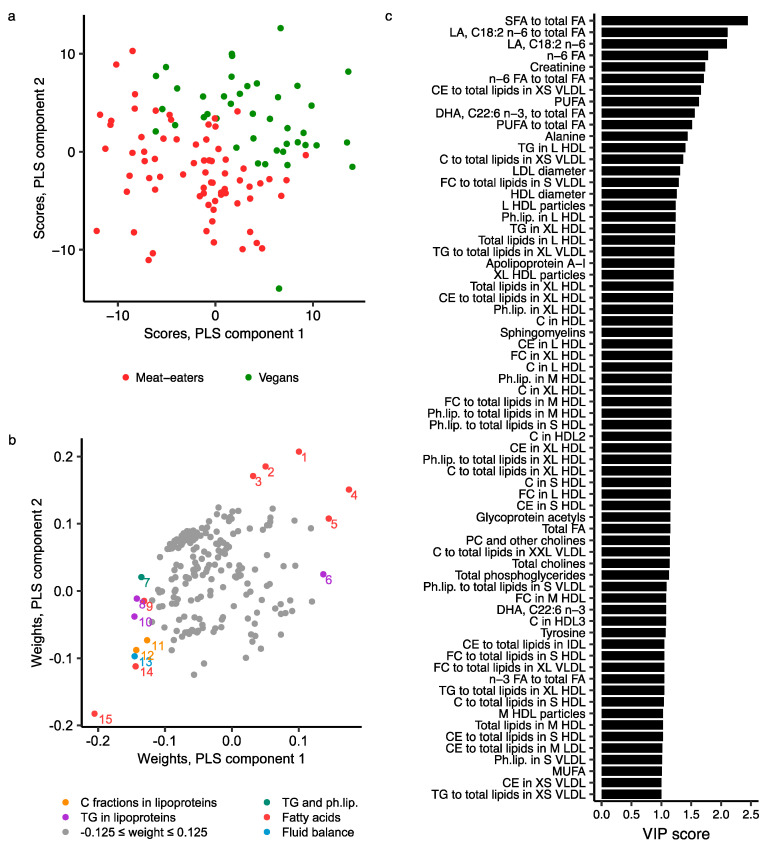
PLS model of 207 NMR metabolite measurements in meat-eaters and vegans. (**a**) Score plot of PLS components 1 and 2 coloured by diet group; (**b**) Weights plot of PLS components 1 and 2. Metabolites with weights more extreme than ±0.125 on either PLS component are coloured and labelled: 1, Linoleic acid, C18:2 n-6; 2, n-6 fatty acids; 3, Polyunsaturated fatty acids; 4, Linoleic acid, C18:2 n-6, to total fatty acids; 5, n-6 to total fatty acids; 6, Triglycerides to total lipids in XL VLDL; 7, Sphingomyelins; 8, Triglycerides in XL HDL; 9, DHA, C22:6 n-3; 10, Triglycerides in L HDL; 11, Total cholesterol to total lipids in XS VLDL; 12, Cholesterol esters to total lipids in XS VLDL; 13, Creatinine; 14, DHA, C22:6 n-3, to total fatty acids; 15, Saturated to total fatty acids; (**c**) Variable influence on projection (VIP) scores, representing the contribution of metabolites to the PLS model; metabolites with VIP > 1 are included in the plot. Further details are given in Table S5. Abbreviations: C, total cholesterol; CE, cholesterol esters; C fractions, cholesterol fractions, i.e., total cholesterol, cholesterol esters and free cholesterols; DHA, docosahexaenoic acid; FA, fatty acids; FC, free cholesterol; HDL, high-density lipoprotein; IDL, intermediate-density lipoprotein; L, large; LA, linoleic acid; LDL, low-density lipoprotein; M, medium; MUFA, monounsaturated fatty acids; Ph.lip., phospholipids; PLS, partial least squares; PUFA, poly unsaturated fatty acids; S, small; SFA, saturated fatty acids; TG, triglycerides; VIP, variable influence on projection; VLDL, very-low-density lipoprotein; XL, extra-large; XS, extra-small; XXL, chylomicrons and extremely large.

When comparing only meat-eaters and vegans (the most extreme groups), the best model included six components and showed higher goodness of fit (R^2^Y = 0.803) and prediction (Q^2^Y = 0.642; Figure S4), than when all four diet groups were included, and also no sign of overfitting (Figure S5). This model explained 83.3% of the total variance in metabolite data. The score plot of components 1 and 2 showed good separation, with vegans having higher scores on both components than meat-eaters (Figure 5a). The metabolites mostly responsible for this separation included fatty acids (mainly ratio of SFA to total fatty acids [highest VIP score of 2.45], absolute concentration and/or ratio to total fatty acids for LA, total n-6 fatty acids, PUFA and DHA), creatinine, lipid fractions in VLDL subtypes (mainly total cholesterol, cholesterol esters and free cholesterol in XS or S VLDL), amino acids (mainly alanine), various lipid-related traits in HDL subclasses (mainly of L and XL HDL), diameter of LDL and HDL particles, apolipoprotein A-I, phospholipids (i.e., sphingomyelins, phosphatidylcholines and other cholines, total cholines, and total phosphoglycerides), and the inflammatory marker glycoprotein acetyls (Figure 5b,c; Table S5).

### 2.4. Comparison of NMR Measures with Those from MS, Clinical Chemistry and GC

The NMR metabolites overlapped with nine analytes obtained using MS (eight amino acids and creatinine), four obtained from clinical chemistry (apolipoproteins and cholesterols) and two analytes obtained from GC (fatty acids).

Between assay-method correlation coefficients (r) for the amino acids and creatinine varied from 0.49 for phenylalanine to 0.94 for alanine (Table 3). For histidine, excluding one extreme outlier reduced the correlation from 0.74 to 0.68. For the apolipoproteins and cholesterols correlations were high (*r* ≥ 0.82), while those for DHA and LA were low (*r* ≤ 0.15).

Inspection of the Bland−Altman plots showed good agreement between the NMR measurements and other measurements at low average concentrations (Figure S6 and Table S6). However, at higher average concentrations, those measured using NMR were lower than those measured using other methods.

**Figure 6:**
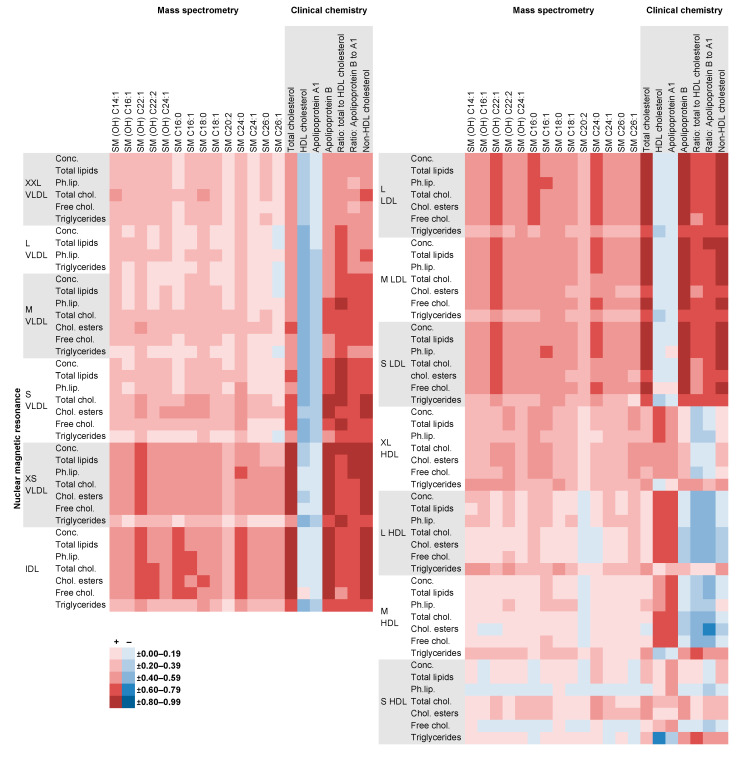
Correlations of NMR lipoproteins with clinical chemistry and MS sphingomyelins. Plasma sphingomyelin concentrations were measured using the mass spectrometry based Absolute*IDQ*^®^ p180 Kit (Biocrates Life Sciences AG, Innsbruck, Austria) [20]. ‘Cx:y’ denotes the number of carbon atoms and double bonds in the fatty acid side chain of the sphingomyelins (SM) and hydroxy-sphingomyelins [SM (OH)]. In serum, apolipoproteins were measured using an immunoturbimetric assay, total cholesterol was measured using an enzymatic assay and HDL cholesterol was measured directly [27]. Further details are seen in Table S7. Abbreviations: Chol., Cholesterol; Conc., concentration; HDL, high-density lipoprotein; IDL, intermediate-density lipoprotein; L, large; LDL, low-density lipoprotein; M, medium; NMR, nuclear magnetic resonance; Ph.lip., phospholipids; S, small; SM, sphingomyelin; SM (OH), hydroxyl-sphingomyelin; VLDL, very-low-density lipoprotein; XL, extra-large; XS, extra-small; XXL, chylomicrons and extremely large.

**Figure 7:**
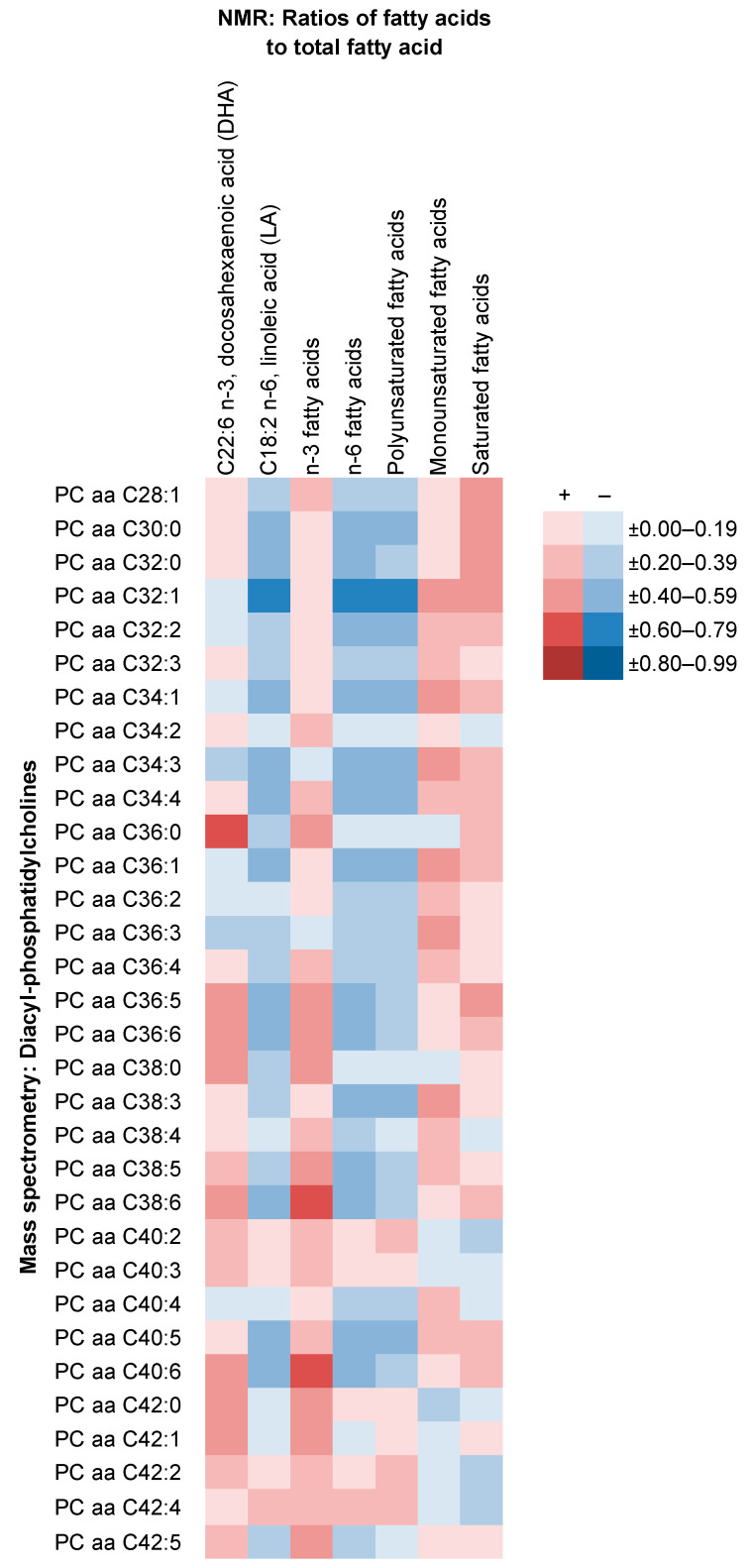
Correlations of NMR fatty acid ratios with MS diacyl-phosphatidylcholine concentrations. Plasma diacyl-phosphatidylcholine (PC aa) concentrations were measured using the mass spectrometry based Absolute*IDQ*^®^ p180 Kit (Biocrates Life Sciences AG, Innsbruck, Austria) [20]. “Cx:y” denotes the total number of carbon atoms and double bonds in the two fatty acid side chains of the phosphatidylcholines. Further details are seen in Table S7.

Correlations of all metabolites measured using NMR with those measured using other methods are shown in Table S7. Correlations ranged from −0.88 (ratio of phospholipids to total lipids in L LDL with apolipoprotein B) to 0.94 (Phospholipids in M LDL with apolipoprotein B). Strong positive correlations were observed especially for NMR lipoprotein subclasses, their lipid concentrations and components with cholesterols measured using clinical chemistry, and for XS VLDL, IDL and LDL subclasses from NMR with some sphingomyelins from MS (Figure 6). Strong inverse correlations were found especially for ratios of free cholesterol, triglycerides and phospholipids to total lipids in lipoprotein subclasses from NMR with total cholesterol, apolipoprotein B and non-high-density lipoprotein (non-HDL) cholesterol from clinical chemistry. The same was the case for the ratios of LA, total n-6 fatty acids and PUFA to total fatty acids with some diacyl-phosphatidylcholines (PC aa; Figure 7).

## 3. Discussion

### 3.1. Main Findings

In this study we compared NMR metabolite measures between four distinct diet groups and compared the NMR measures to those from a MS-based metabolomics platform, clinical chemistry and GC.

In the analysis of differences in metabolite profiles between diet groups, we consistently found differences for fatty acids. Vegans had higher levels (i.e., concentrations and/or ratios to total fatty acids) of LA, total n-6 fatty acids and PUFA, and lower levels of DHA, total n-3 fatty acids and SFA, compared to meat-eaters. This is in accordance with previous results from this cohort [28], with other data [33] and with the observed intakes of these fatty acids (Table 1) [33]. For total n-3 fatty acids and DHA measures, vegetarians had lower levels similar to those of vegans and fish-eaters had higher levels similar to those of meat-eaters. For the other fatty acids, vegetarians and fish-eaters mostly had intermediate levels. Moreover, consistently lower concentrations of sphingomyelins, tyrosine and creatinine, and particle diameter of LDL and HDL were observed in vegans, as were higher concentrations of alanine; for all but the amino acids, meat-eaters had the highest levels, followed by fish-eaters and vegetarians. Finally, many differences were observed for various lipid fractions in VLDL, including triglycerides and cholesterols (for which concentrations were lowest in vegans), and in HDL (for which concentrations were mostly lowest in vegans). The results for HDL are in line with those previously reported in this cohort for total HDL cholesterol [27].

While the agreement between measurements of the overlapping metabolites on the NMR platform with the MS- and clinical chemistry-based methods was high for alanine, isoleucine, leucine, tyrosine, creatinine, apolipoproteins, total and HDL cholesterols (*r* ≥ 0.83; in line with other data [15,34]), it was lower for the remaining amino acids (*r* ≥ 0.49). The very low correlations for DHA and LA concentrations (*r* ≤ 0.15) observed here are in contrast with other studies comparing the NMR protocol with gas chromatography (*r* ≥ 0.95) [14,35]. However, the associations of DHA and LA measured using NMR with diet group in the current analysis were comparable with those previously observed in EPIC-Oxford [28] and elsewhere [33] (both GC), although less strong. More widely, strong correlations were observed between the metabolites covered by the NMR platform and other assay methods. This was especially the case for NMR lipoprotein subclasses and their lipid concentrations and compositions with lipids measured using clinical chemistry (e.g., apolipoprotein B), suggesting that standard clinical chemistry may capture a substantial proportion of the information provided by the NMR platform for the lipoproteins. Strong correlations were also observed for NMR lipoprotein subclasses with MS-based sphingomyelins, and for NMR metabolite measures involving PUFA with diacyl-phosphatidylcholines from MS.

### 3.2. Findings in Context of the Literarure 

While some studies have compared metabolomics platforms commonly used in prospective studies using split samples [29,36,37,38], to our knowledge none has included both the Biocrates (MS) and Nightingale (NMR) platforms (both commonly used in prospective studies of metabolomics and disease risk [29]), as we do here. As shown, there was limited overlap between the metabolites measured by these two platforms. However, for creatinine and the eight amino acids which did overlap, we observed good to excellent between-method correlations and consistent findings by diet group between the current and our previous studies [20,21]. 

Differences in coverage of the metabolome by different metabolomics platforms make comparisons of results across metabolomics studies complex. Moreover, for studies of metabolite profiles by diet group, study populations differ and dietary habits within the groups are likely also to differ. However, here we compare our current findings to previous published results. One previous study has compared the blood NMR metabolite profile of diet groups, including omnivores, fish-eaters, vegetarians and vegans, as in the current study (*n*_total_ = 120) [23]. In contrast to our study, Lindqvist et al. included both men and women (62.5%), used more standardised blood collection and handling procedures (including fasting samples and controlled temperature), and used another NMR technology, which measured fewer metabolites (n~70). The main findings included lower isoleucine, leucine, valine and creatine, and higher glutamine, glycine and trimethylamine in vegans compared to non-vegans. Moreover, creatinine was higher in meat-eaters than in non-meat-eaters; total cholesterol, LDL, HDL and creatinine measured using clinical chemistry were also highest in the meat-eaters [23]. The findings for leucine, valine, glutamine, creatinine and cholesterols were in line with the current study and with our study of metabolites measured using the MS-based Biocrates platform [20]. Similarly, a small study (*n* = 36), by Wang et al., comparing 66 MS metabolites in fasted serum between three diet groups, has also reported lower concentrations of leucine and valine, as well as of isoleucine, in vegans and vegetarians compared to omnivores [24]. Glycine was not measured by the Nightingale platform or by Wang et al. [24] but Lindqvist et al.’s results [23] correspond to our previous findings [20,21]. Trimethylamine and creatine were not measured by the platforms from Nightingale or Biocrates, nor by Wang et al. [24]. Finally, while Lindqvist et al. reported that lipids (reported as one trait) were not a major contributor to the separation between diet groups, in models including men [23], various lipids (including cholesterols, fatty acids, phospholipids and triglycerides) were important for group separation in the current study, in the study by Wang et al. [24], and in our previous MS-based study [20]. Specifically, a key finding of our previous study was lower concentrations of numerous phosphatidylcholines and sphingomyelins in vegans, which were the metabolites mostly responsible for separation between diet groups [20]. In the NMR assay, these two metabolite classes were measured as part of the phospholipid components of lipoprotein subclasses and as part of total phosphoglycerides, total cholines, phosphatidylcholine and other cholines, and sphingomyelins. While concentrations of these metabolites were consistently lower in vegans, they did not have a major influence on the discrimination between diet groups in the current study.

Although we did not aim to investigate the possible mediating role of metabolites in associations between diet groups and disease outcomes, our results on NMR metabolites may help to interpret studies of the associations of diet groups with disease risk. For example, prospective studies have reported lower risk of type 2 diabetes in vegans [9,10] and in individuals with higher circulating LA [39,40]. We found that vegans had higher LA levels than the other diet groups, and thus circulating LA could play a role in the association between diet group and risk of type 2 diabetes, albeit with BMI as a major contributing factor. Furthermore, several ratios of fatty acids to total fatty acids, including DHA, total n-3, LA, total n-6, SFA and PUFA, which were among the metabolite measures most strongly associated with diet group in the current study, have been linked to lower risk of cardiovascular events [14,15,16]. In the current study, some of these fatty acids ratios were lowest in vegetarians and vegans (DHA and total n-3), possibly suggesting a higher risk of cardiovascular disease in these diet groups, while other were highest in vegans followed by fish-eaters (LA, total n-6 and PUFA), suggesting a lower risk in these groups. This might possibly be in line with the lower risk of ischemic heart disease observed in fish-eaters, and especially in vegetarians and vegans, compared to meat-eaters [4]. However, to understand the potential mediating role of these fatty acids ratios in the associations between diet group and subtypes of cardiovascular disease, further studies are needed.

### 3.3. Strengths and Limitations

The main strengths of this study are the inclusion of four distinct diet groups from a well characterized cohort, which allowed a detailed study of metabolite profile of various diets, while limiting the potential role of confounding. Moreover, we investigated two aspects important for planning and interpreting studies of metabolites in relation to disease risk and diet-disease associations. We were able to describe the largest study of differences in NMR metabolites, the first for the Nightingale platform, and to provide the first comparison of metabolites measured using the Nightingale and the Biocrates (MS) platforms. 

There are however limitations to the study. Samples were non-fasted and were not cooled during transport to the laboratory, which may have affected the results of the NMR metabolomics assay [41,42], albeit studies exploring the potential impact of pre-processing conditions on the specific metabolites measured using the Nightingale platform are scarce. One study has reported that most of the metabolites measured using the Nightingale platform in non-fasted serum samples were not materially affected by delaying processing for 48 h at 21 °C (experimental condition; conditions similar to those in the current study) compared to processing within 1.5 h at 4 °C (reference condition) [42]. For metabolites consistently showing up as different between diet groups in the current analysis, rank correlations between metabolite concentrations in samples kept at the experimental versus the reference condition were 0.7 or above, except for, tyrosine (*r* = 0.6) and lipid traits in small HDL (ranging from 0.6 and 0.7). Moreover, in the current analysis, fasting status and time from blood collection to processing explained only a small proportion (0.6% and 1.5%, respectively) of the total variability of NMR metabolites. Fasting status did not differ greatly between diet groups, but some differences were observed for process delay. In combination, this suggests that bias arising from pre-processing did not have much impact on our results. 

A further limitation was the lack of data on the type of medication and supplements taken on the day of blood collection. We found that any use explained 3.9% of the total variability of the NMR metabolite measures and that the proportion of participants taking any medication or supplements did not materially differ between diet groups. It has been shown that starting statin treatment is associated with changes in the measured lipoproteins concentrations and components, apolipoproteins, fatty acid concentrations and ratios, while metabolites in other classes were mainly unrelated to statin use [43]. Finally, the NMR and MS assays were done on serum and plasma samples, respectively, which may have impacted the comparability of metabolite levels measured using the two platforms [44].

### 3.4. Future Work

While this study provides insights into the comparability and complementarity of two metabolomics platforms and other assay methods, comparisons using split samples with other platforms frequently used for metabolomics studies in epidemiology are also warranted [29]. Moreover, further research on the effect of factors related to blood sample collection, handling and storage on the metabolites measured using the Nightingale NMR platform would aid interpretation of large-scale cohort data on these metabolites and disease risk. Finally, many epidemiological studies rely on a single metabolite measurement to represent long-term exposure in analyses of disease risk. To our knowledge, one study has reported poor to excellent reproducibility of the metabolites measured using the Nightingale platform in fasting serum samples over 3.6 years (median intra-class correlation coefficient of 0.66, range 0.17 to 0.84) [45]; replication of these results and reproducibility over longer time periods would also provide valuable information for studies of these metabolites and disease outcomes.

## 4. Materials and Methods 

### 4.1. Study Population and Data Collection

EPIC-Oxford consists of 65,000 men and women aged at least 20 years who were recruited across the United Kingdom between 1993 and 2000 [46]. The main aim of this cohort is to investigate associations between diet, lifestyle and cancer risk in individuals with different long-term dietary habits; thus, a large number of non-meat-eaters were recruited.

At recruitment, participants provided extensive information on diet, lifestyle, body size and previous disease, and 20,000 also provided a blood sample. The diet groups were defined as meat-eaters (who eat meat), fish-eaters (who eat fish but not meat), vegetarians (who eat neither meat nor fish) and vegans (who do not eat meat, fish, dairy or eggs) based on “yes”/“no”-questions on intakes of meat, fish, dairy products or eggs. Moreover, participants filled in a 130-item validated semi-quantitative food frequency questionnaire [47,48]. Blood sample were taken at the participant’s local general practitioner’s surgery; participants were not required to fast. Whole blood was transported by post at ambient temperature to the laboratory where they were processed for long-term storage in liquid nitrogen (−196 °C) until 2011 and subsequently in electric freezers (−80 °C).

Participants in the current study were a subset of participants in our previous study of MS-based metabolites by diet group [20]. In brief, all participants were men between 30 and 49 years of age, with known smoking status and diet group, with reliable energy intake, and without prior major disease.

All participants gave written informed consent prior to entering the EPIC-Oxford study. The study was conducted in accordance with the Declaration of Helsinki, and the protocol for EPIC-Oxford was approved by a Multicentre Research Ethics Committee (MREC/02/0/90): Scotland A Research Ethics Committee (IRAS 223031; updated approval on 28/03/2018).

### 4.2. Laboratory Analysis

A total of 225 metabolite measures, including 146 fully quantified metabolite concentrations and 79 derived variables, were obtained from serum samples using a targeted high-throughput NMR spectroscopy platform (Nightingale Health Ltd., Helsinki, Finland) [49,50,51]. This method provides simultaneous quantification of common lipids, lipoprotein subclass profiling with lipid concentrations within 14 subclasses, fatty acid composition, and various low-molecular weight metabolites including amino acids, ketone bodies and markers of glycolysis, fluid balance and inflammation (an overview of the spectral region of the metabolites is available in [49]). A detailed description of the sample preparation and acquisition parameters has been published previously [49,50]. 

In brief, the frozen samples are thawed overnight in a fridge at +4 °C, mixed, and centrifuged at 3400× *g* for 10 min. Thereafter, 70 µL serum was mixed with 70 µL sodium phosphate buffer (75 mM Na_2_HPO_4_ in 80%/20% H_2_O/D_2_O, pH 7.4; including also 0.08% sodium 3-(trimethylsilyl)propionate-2,2,3,3-d4 and 0.04% sodium azide) for a total sample volume of 140 µL in 3 mm outer diameter SampleJet NMR tubes. This sample preparation was done using an automated procedure (PerkinElmer JANUS Automated Workstation, Waltham, MA, USA). No extraction procedure was applied. The samples were then preheated to 37.5 °C just prior to spectra acquisition, which was performed using a Bruker AVANCE III spectrometer operating at 500.36 MHz (1H observation frequency, 11.74 T; Bruker, MA, USA). Data were acquired using two NMR pulse sequences. Lipids and macromolecular data were acquired using a standard Bruker NOESY PRESAT (noesygppr1d) sequence. This was acquired with 80k data points using 8 transients after 4 dummy scans. The 90° pulse length was automatically calibrated for each sample, the mixing time was 10 ms, and an irradiation field of 25 Hz was used to suppress the water peak. The acquisition time was 2.7 s and the relaxation delay 3.0 s. Low molecular weight metabolite data were acquired using a standard Bruker CPMG (cpmgpr1d) sequence. This was acquired using 64k data points using 16 transients after 4 dummy scans. The same automatically calibrated pulse length as in the NOESY was used for each sample, the CPMG T2-filter was 78 ms with a 403 µs fixed-echo delay, and an irradiation field of 25 Hz was used to suppress the water peak. The acquisition time was 3.3 s and the relaxation delay 3.0 s. All free induction decays were phase corrected and processed in an automated fashion. The FIDs were zero-filled to 128k data points and then an exponential window function with 1.0 Hz line broadening was applied.

We included 9% (*n* = 35) quality control samples of pooled plasma among the study samples. Laboratory personnel were blinded to diet group and quality control sample status. Metabolites with coefficients of variation from the blinded quality control samples greater than 20% were excluded (*n* = 15; Table S8 and Figure S1). Furthermore, we excluded metabolites for which more than 20% of men had non-quantifiable measurements (missing or below the limit of quantification [LOQ]); *n* = 1). Metabolite values below the LOQ were set to half the lowest measured value for each metabolite. Finally, we excluded glucose and lactate because glucose concentrations below physiological levels accompanied by high lactate concentrations were detected in 64.4% of samples; this indicates glycolysis after blood collection, probably due to the time in post of whole blood before separating plasma from cell-containing blood fractions. Thus, 207 metabolite variables, including 75 ratios, were included in the statistical analysis.

Samples from 287 men were analysed at Nightingale Health, Helsinki, Finland (Figure S1). We excluded one participant (vegetarian), for whom 80.9% of metabolites had non-quantifiable values, leaving 286 men for the statistical analysis.

From three previous studies using split samples from the same baseline blood collection as for the NMR assay, we have MS-based plasma metabolite concentrations (Absolute*IDQ*^®^ p180 Kit, Biocrates Life Sciences AG, Innsbruck, Austria) [20], standard clinical chemistry measurements of serum total cholesterol, non-HDL-cholesterol, HDL cholesterol, and apolipoproteins A–I and B [27], and GC measurements of plasma fatty acids DHA and LA [28], for some or all participants in the current study.

### 4.3. Statistical Analysis

Before analyses, all metabolite variables were logarithmically transformed to approximate the normal distribution. Pearson correlations between pairs of NMR metabolites were calculated.

To investigate the contribution of covariates (i.e., diet group, participant characteristics and factors related to blood collection and handling) to the variability in the NMR metabolite measures, we used the PC-PR2 method [32]. This method quantifies the proportion of the total variability in metabolite measures which is explained by each covariate after adjusting for all other covariates in the model.

Differences in geometric mean concentrations and ratios of metabolites across diet group were tested by ANOVA, adjusting for age (30–34, 35–39, 40–44, 45–49 years), BMI (<22.5, 22.5–24.9, ≥25 kg/m^2^, unknown), smoking (never, former, current), alcohol intake (<1, 1–7, 8–15, ≥16 g/d), physical activity level (inactive, moderately inactive, moderately active, active, unknown), time since last meal at blood collection (<1.5, 1.5–2.9, 3.0–4.4, ≥4.5 h, unknown) and time from blood collection to processing (quartiles of the distribution, i.e., <24.5, 24.6–41.3, 41.4–71.6, ≥71.7 h, unknown). We controlled for multiple testing using the Benjamini-Hochberg false discovery rate (FDR) method with a significance level of 0.05 [52]. Due to the differences in units for the NMR metabolites, we repeated the analysis after z-score standardising (mean = 0 and SD = 1) the log-transformed metabolite variables. In this analysis, we standardised to the adjusted mean in meat-eaters to provide an estimate of the difference from each of the non-meat-eating diet groups to the meat-eaters. 

To explore latent patterns in metabolite profiles, and for dimension-reduction, we conducted multivariate analyses. For these analyses we excluded men with missing data on one or more of the 207 included metabolite measures (*n* = 49), leaving 237 men in the analyses. Overall z-score standardised log-transformed metabolite variables were used for these analyses. 

First, we conducted a PCA, which maximises the variance explained in metabolite levels [53]. The relationship between the derived latent patterns in metabolite profile (principal components) and diet group was assessed using score plots. 

Second, to obtain patterns in metabolite profile best differentiating diet groups, we ran PLS regression, which only accounts for metabolite information which is correlated with diet group [13] by maximising the covariance of the relationship between metabolite variables and diet group [53]. The number of components to retain was determined by assessing the goodness of fit (R^2^Y) and goodness of prediction (Q^2^Y), derived using seven-fold cross-validation; the algorithm identifies where the increase in Q^2^Y per additional component retained plateaus [53,54]. To assess the potential overfitting of the PLS models, we used permutations tests (*n* = 200), in which the assignment of diet group was random; higher goodness of fit and prediction for the actual model than the permutation models and negative values for goodness of prediction of the permutation models indicate validity of the actual model. We assessed which metabolites were most influential for the model using PLS weights for each retained component and VIP scores across all retained PLS components. The mean squared VIP is 1 and thus a VIP > 1 is commonly used as a cut-off to denote important metabolites [53,54]. Two PLS models were run; one using all four diet groups and another including only meat-eaters and vegans (*n* = 112).

For metabolites measured using both NMR and one of the other methods, we compared the concentrations using Pearson correlations and Bland–Altman plots. Moreover, we calculated Pearson correlations between all metabolites measured using NMR and each of the other assay method.

We used R (Foundation for Statistical Computing, Vienna, Austria. URL: https://www.R-project.org/, accessed on 27 January 2021) for the statistical analyses of PC-PR2, PCA and PLS (‘ropls’ package for PCA and PLS). The remaining analyses were run using Stata Statistical Software Package, version 15 (Stata Corporation, College Station, TX, USA).

## 5. Conclusions

The metabolomics profiles of male meat-eaters, fish-eaters, vegetarians and especially vegans are different. This is mainly due to differences in fatty acids (lower levels of DHA, total n-3 fatty acids and SFA, and higher levels of LA, total n-6 fatty acids and PUFA in vegans compared to meat-eaters), and lower levels of cholesterol and triglycerides in VLDL, of various lipid fractions in HDL subclasses, and of sphingomyelins, tyrosine and creatinine, as well as higher alanine in vegans. Levels in fish-eaters and vegetarians differed by analyte but for many metabolite measures, the levels in fish-eaters and vegetarians were intermediate to those of meat-eaters and vegans.

There was moderate to strong comparability of the few specific metabolite concentrations measured both using NMR, and MS or clinical chemistry. Moreover, many strong correlations were found across all measured metabolites from NMR versus MS and especially clinical chemistry. Thus, the studied NMR and MS platforms are complementary.

## Figures and Tables

**Table 1:** Participant characteristics, nutrient intakes and factors related to blood collection and handling by diet group ^1^.

	Meat-Eaters (*n* = 80)	Fish-Eaters (*n* = 69)	Vegetarians (*n* = 74)	Vegans (*n* = 63)
Participant characteristics				
Age at blood collection, years	44.0 (37.0, 44.0)	43.0 (38.0, 46.0)	44.0 (36.0, 44.0)	42.0 (38.0, 46.0)
Body mass index ^2^, kg/m^2^	24.5 (22.1, 26.1)	22.7 (21.1, 24.4)	22.9 (21.6, 25.7)	22.1 (20.5, 24.4)
Waist circumference ^2^, cm	86.0 (81.0, 91.0)	81.0 (79.0, 86.0)	84.0 (81.0, 86.0)	81.0 (76.0, 89.0)
Current smoker	10 (12%)	6 (9%)	5 (7%)	3 (5%)
Very physically active ^2,3^	19 (25%)	12 (18%)	11 (16%)	21 (35%)
Alcohol intake, g/d	9.8 (3.2, 17.5)	10.6 (4.9, 30.0)	10.6 (5.1, 28.6)	2.9 (1.0, 13.0)
Nutrient intakes ^4^				
Energy, kJ/d	9562 (8156, 11508)	9681 (8347, 11025)	9640 (8197, 11523)	8169 (6615, 9689)
Protein, %E	14.0 (12.7, 15.7)	13.0 (11.6, 14.6)	12.6 (11.1, 13.9)	12.6 (11.2, 13.8)
Carbohydrate, %E	51.5 (47.9, 55.7)	51.8 (49.0, 58.4)	53.9 (48.8, 58.1)	58.8 (54.9, 64.0)
Total fat, %E	31.6 (28.3, 34.8)	31.9 (26.6, 35.4)	31.2 (28.7, 34.9)	28.6 (22.9, 33.9)
SFA, %E	10.2 (8.7, 12.1)	9.7 (7.7, 11.8)	9.8 (8.0, 11.8)	5.4 (4.2, 6.8)
MUFA, %E	11.0 (9.1, 11.9)	10.2 (8.9, 11.7)	10.7 (9.2, 11.9)	10.7 (7.8, 12.2)
PUFA, %E	7.2 (6.2, 8.1)	7.9 (6.8, 8.8)	8.0 (6.8, 9.1)	9.6 (7.9, 11.4)
DHA (C22:6 n-3), %E	0.031 (0.021, 0.044)	0.028 (0.020, 0.043)	0.003 (0.002, 0.005)	-
LA (C18:2 n-6), %E	6.4 (5.5, 7.1)	7.0 (6.0, 7.8)	7.0 (6.0, 8.2)	8.3 (6.8, 9.6)
Blood sample related factors				
Time since last meal ^2^, h	1.8 (1.0, 3.3)	2.0 (1.2, 4.0)	2.3 (1.5, 4.0)	2.5 (1.5, 4.1)
Meds./supplements taken ^2^	54 (68%)	44 (65%)	51 (69%)	40 (63%)
Time, collection ^2^, hh:mm	11:10 (10:00, 15:20)	10:50 (9:45, 15:38)	10:25 (9:40, 13:00)	10:30 (9:35, 15:00)
Process delay ≤ 32 h ^2,5^	52 (66%)	24 (35%)	37 (51%)	27 (46%)

^1^ Values are median (inter-quartile range) and n (%) for continuous and categorical variables, respectively.^2^ Information was missing for some participants; percentages or medians were calculated without these. ^3^ Defined according to a modified version of the Cambridge Physical Activity Index [30]. ^4^ All nutrient intakes were estimated using the National Nutrient Database for Standard Reference of the United States, as described earlier [31], and may not exactly match those previously reported for this study population [20], which were estimated using the McCance and Widdowson’s The Composition of Foods. ^5^ Samples were sent by post to the laboratory; process delay ≤ 32 h corresponds to samples which arrived by the end of the next day. Abbreviations: DHA, docosahexaenoic acid (C22:6 n-3); LA, linoleic acid (C18:2 n-6); Meds., medication; MUFA, monounsaturated fatty acids; PUFA, polyunsaturated fatty acids; SFA, saturated fatty acids; %E, percent of energy.

**Table 2:** Geometric mean of selected NMR metabolites by diet group on the original scale ^1^.

Metabolites	Meat-Eaters (*n* = 80)	Fish-Eaters (*n* = 69)	Vegetarians (*n* = 74)	Vegans *(n* = 63)	*p(adj)* ^2^
	Mean (95% CI)	Mean (95% CI)	Mean (95% CI)	Mean (95% CI)	
XS VLDL, μmol/L					
Cholesterol esters	159 (151, 168)	154 (145, 162)	149 (141, 157)	132 (124, 140)	6 × 10^−^^4^
XL HDL, μmol/L					
Triglycerides	11.4 (9.95, 13.1)	11.1 (9.62, 12.8)	11.2 (9.85, 12.8)	6.89 (5.93, 8.00)	3 × 10^−^^5^
L HDL, μmol/L					
Triglycerides	20.3 (17.9, 23.0)	20.4 (18.0, 23.2)	20.1 (17.8, 22.7)	12.9 (11.3, 14.8)	2 × 10^−^^5^
Ratio to total lipids in XXL VLDL, %					
Total cholesterol ^3^	17.1 (15.9, 18.3)	18.0 (16.7, 19.3)	17.2 (16.1, 18.4)	13.6 (12.6, 14.8)	6 × 10^−^^5^
Ratio to total lipids in XL VLDL, %					
Triglycerides ^3^	60.2 (59.0, 61.3)	61.0 (59.8, 62.3)	62.6 (61.5, 63.7)	64.5 (63.0, 66.1)	7 × 10^−^^4^
Ratio to total lipids in M VLDL, %					
Total cholesterol	28.6 (27.8, 29.3)	28.4 (27.7, 29.2)	28.0 (27.3, 28.8)	26.2 (25.5, 27.0)	8 × 10^−^^4^
Triglycerides	50.8 (50.1, 51.5)	51.1 (50.4, 51.8)	51.6 (51.0, 52.3)	53.1 (52.3, 53.8)	8 × 10^−^^4^
Ratio to total lipids in XS VLDL, %					
Total cholesterol	49.4 (48.9, 50.0)	49.1 (48.6, 49.6)	48.6 (48.2, 49.1)	47.3 (46.7, 47.8)	8 × 10^−^^6^
Cholesterol esters	33.6 (33.1, 34.1)	33.3 (32.9, 33.8)	33.1 (32.7, 33.6)	31.5 (31.0, 31.9)	3 × 10^-7^
Ratio to total lipids in IDL,%					
Phospholipids	27.5 (27.3, 27.6)	27.5 (27.3, 27.7)	27.5 (27.4, 27.7)	60.2 (59.6, 60.8)	1 × 10^−^^3^
Cholesterol esters	43.9 (43.5, 44.4)	43.7 (43.3, 44.1)	44.1 (43.7, 44.5)	42.6 (42.2, 43.1)	4 × 10^−^^4^
Ratio to total lipids in M HDL, %					
Phospholipids	47.1 (46.7, 47.5)	47.2 (46.8, 47.6)	46.8 (46.4, 47.1)	46.0 (45.6, 46.4)	6 × 10^−^^4^
Triglycerides and phospholipids, μmol/L					
Sphingomyelins ^3^	391 (378, 405)	376 (363, 389)	366 (354, 378)	339 (327, 352)	3 × 10^−^^5^
Fatty acids, μmol/L					
Estimated degree of unsaturation^3^	1.19 (1.18, 1.19)	1.17 (1.16, 1.18)	1.15 (1.14, 1.16)	1.17 (1.16, 1.18)	9 × 10^−^^6^
DHA, C22:6 n-3 ^3^	126 (118, 134)	124 (116, 132)	99.3 (93.4, 106)	97.5 (91.0, 104)	2 × 10^−^^9^
n-3 fatty acids ^3^	380 (355, 407)	376 (350, 403)	312 (292, 334)	307 (285, 331)	2 × 10^−^^5^
Ratios of fatty acids to total fatty acid, %					
DHA, C22:6 n-3 ^3^	1.27 (1.21, 1.33)	1.27 (1.21, 1.33)	1.01 (0.96, 1.05)	1.03 (0.98, 1.09)	2 × 10^−^^13^
LA, C18:2 n-6 ^3^	28.3 (27.5, 29.1)	30.1 (29.2, 30.9)	29.1 (28.3, 29.9)	31.9 (30.9, 32.9)	3 × 10^−^^5^
n-3 fatty acids ^3^	3.82 (3.65, 4.00)	3.85 (3.67, 4.03)	3.16 (3.03, 3.31)	3.25 (3.09, 3.42)	5 × 10^−^^10^
n-6 fatty acids ^3^	33.5 (33.0, 34.1)	34.3 (33.7, 34.9)	33.5 (33.0, 34.1)	35.5 (34.8, 36.1)	6 × 10^−^^4^
Polyunsaturated fatty acids ^3^	37.4 (36.8, 38.0)	38.2 (37.6, 38.9)	36.7 (36.2, 37.3)	38.8 (38.1, 39.5)	8 × 10^−^^4^
Saturated fatty acids ^3^	36.3 (36.0, 36.6)	36.0 (35.7, 36.3)	36.3 (36.0, 36.6)	34.7 (34.4, 35.1)	5 × 10^−^^10^
Amino acids, μmol/L					
Tyrosine ^3^	54.8 (52.5, 57.1)	57.2 (54.8, 59.7)	59.1 (56.7, 61.6)	51.1 (48.8, 53.5)	6 × 10^−^^4^

^1^ All analyses were adjusted for age, BMI, smoking, alcohol, physical activity level, time since last meal at blood collection and time from blood collection to processing.^2^ *p(adj)* is the *p*-value after adjusting for multiple testing using a FDR of 0.05 on all 207 metabolite variables. Metabolite measures with *p(adj)* < 0.001 are shown. Results for all NMR metabolites are shown in Table S2. ^3^ Missing values for some participants. Abbreviations: CI, confidence interval; DHA, docosahexaenoic acid; HDL, high-density lipoprotein; IDL, intermediate-density lipoprotein; L, large; LA, linoleic acid; M, medium; SD, standard deviation; VLDL, very-low-density lipoprotein; XL, extra-large; XS, extra-small; XXL, chylomicrons and extremely large.

**Table 3:** Pearson correlations between NMR metabolite concentrations and those measured using another method.

Metabolites	*n*	*r*	*p*
Mass spectrometry ^1^			
Alanine	286	0.94	<0.0001
Glutamine	279	0.62	<0.0001
Histidine	284	0.74	<0.0001
Histidine, excl. 1 outlier	283	0.68	<0.0001
Isoleucine	286	0.83	<0.0001
Leucine	286	0.87	<0.0001
Valine	286	0.70	<0.0001
Phenylalanine	220	0.49	<0.0001
Tyrosine	284	0.87	<0.0001
Creatinine	284	0.84	<0.0002
Clinical chemistry ^2^			
Apolipoprotein A-I	286	0.82	<0.0001
Apolipoprotein B	286	0.92	<0.0001
Total cholesterol	286	0.94	<0.0001
Total high-density lipoprotein (HDL) cholesterol	286	0.86	<0.0001
Capillary gas-liquid chromatography ^3^			
C22:6 n-3, docosahexaenoic acid (DHA)	71	0.03	0.8
C22:6 n-3, docosahexaenoic acid (DHA), excl. 1 outlier	70	−0.01	0.9
C18:2 n-6, linoleic acid (LA)	72	0.15	0.2

^1^ Plasma amino acid and creatinine concentrations were measured using the mass spectrometry based Absolute*IDQ*^®^ p180 Kit (Biocrates Life Sciences AG, Innsbruck, Austria) [20]. ^2^ In serum, apolipoproteins were measured using an immunoturbimetric assay, total cholesterol was measured using an enzymatic assay and HDL cholesterol was measured directly [27]. ^3^ Total plasma esterified and nonesterified fatty acids were measured using capillary gas-liquid chromatography [28].

## Data Availability

The data access policy for the EPIC-Oxford study is available via the study website (www.epic-oxford.org/data-access-sharing-and-collaboration/ (accessed on 20 February 2021)).

## Supplementary Materials

The following are available online at https://www.mdpi.com/2218-1989/11/2/121/s1, Figure S1. Exclusion of men and NMR metabolite measures, Figure S2. Cumulative goodness of fit (R2Y) and goodness of prediction (Q2Y) by the retained components (p1–p7) for the partial least squares model of 207 NMR metabolite measures in four diet groups, Figure S3. Validation of the partial least squares model of 207 NMR metabolite measures in four diet groups, Figure S4. Cumulative goodness of fit (R2Y) and goodness of prediction (Q2Y) by the retained components (p1–p6) for the partial least squares model of 207 NMR metabolite measures in meat-eaters and vegans, Figure S5. Validation of the partial least squares model of 207 NMR metabolite measures in meat-eaters and vegans, Figure S6. Bland–Altman plots comparing NMR metabolite concentrations to those measured using another method, Table S1. Pearson correlations between pairs of NMR metabolite measures in 286 men, Table S2. Geometric mean NMR metabolite measures by diet group on the original scale, Table S3. Geometric mean NMR metabolite measures by diet group standardised to the adjusted mean in meat-eaters, Table S4. Loadings from principal component analysis of four diet groups, Table S5. Weights and variable influence on projections (VIP) scores for the partial least squares models of 207 NMR metabolite measures in four diet groups and in meat-eaters and vegans, respectively, Table S6. Bland–Altman estimates of NMR metabolite concentrations compared to those measured using other methods, Table S7. Pearson correlations between NMR metabolite measures and metabolites measured using other methods in 286 men, Table S8. Completeness and coefficients of variation of metabolite measures from the NMR platform, and exclusion of metabolites for statistical analyses.
