# Supplementary material for: NMR Metabolite Profiles in Male Meat-Eaters, Fish-Eaters, Vegetarians and Vegans, and Comparison with MS Metabolite Profiles
Source: Metabolites. 2021 Feb 20;11(2):121. doi: 10.3390/metabo11020121 (PMC7923783; doi:10.3390/metabo11020121)
Supplement: Supplementary file 1 [file metabolites-11-00121-s001.zip › metabolites-1107316-supplementary/Supplemenraty_Figures_Matebolites_v2_proofs.docx]

Schmidt JA et al. NMR metabolite profiles in male meat-eaters, fish-eaters, vegetarians and vegans, and comparison with MS metabolite profiles. Metabolites 2021

**Supplementary Figures**

# Figure S1. Exclusion of men and NMR metabolite measures ………………………………..………………. 2

Figure S2. Cumulative goodness of fit (R2Y) and goodness of prediction (Q2Y) by the retained components (p1-p7) for the partial least squares model of 207 NMR metabolite measures in four diet groups………………………………………………………………………………………………………………… 3

Figure S3. Validation of the partial least squares model of 207 NMR metabolite measures in four diet groups………………………………………………………………………………………………………………… 4

Figure S4. Cumulative goodness of fit (R2Y) and goodness of prediction (Q2Y) by the retained components (p1-p6) for the partial least squares model of 207 NMR metabolite measures in meat-eaters and vegans………………………………………………………………………………………………………….. 5

Figure S5. Validation of the partial least squares model of 207 NMR metabolite measures in meat-eaters and vegans…………………………………………………………………………………………………………... 6

Figure S6. Bland–Altman plots comparing NMR metabolite concentrations to those measured using another method……………………………………………………………………………………………………… 9

**Exclusions of metabolites**

Degraded metabolites
n=2

Metabolites
n=209 incl. 75 derived

Metabolites
n=210 incl. 75 derived

Metabolites obtained
n=225 incl. 79 derived

Non-quantifiable measurements >20%
n=1

Blinded coefficient of variation > 20%
n=15

Metabolite in analysis n=207 incl. 75 derived

**Exclusions of participants**

Measurements in univariate analysis, n=286

Measurements in multivariate analysis, n=237

Measurements from participants
n=287

Total samples measured
n =317

No value for ≥1 of 207 metabolites
n=49

Non-quantifiable measurement for 182 of 225 metabolites, n=1

Blinded quality control samples
n=30

# Figure S1. Exclusion of men and NMR metabolite measures

Measurements in univariate analysis, n=286

Measurements in multivariate analysis, n=237

Valid measurements n=287

Measurements from participants
n=375

Measurements obtained
n=410

No value for ≥1 of 207 metabolites
n=49

Non-quantifiable measurement for 182 of 225 metabolites, n=1

Measurements failed
n=88

Quality control samples
n=35

**Exclusions of participants**

2

**
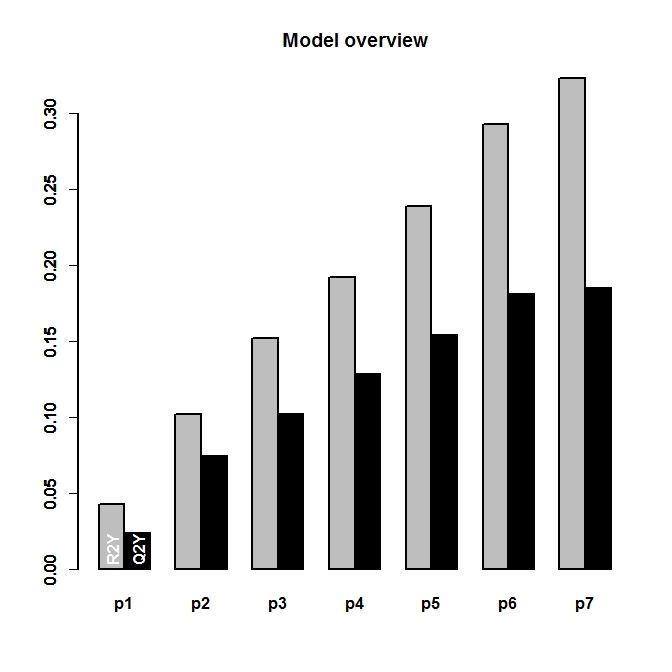
**Figure S2. Cumulative goodness of fit (R2Y) and goodness of prediction (Q2Y) by the retained components (p1-p7) for the partial least squares model of 207 NMR metabolite measures in four diet groups.

Seven-fold cross validation was used. R2Y = 0.323 and Q2Y = 0.185.

**3**

**
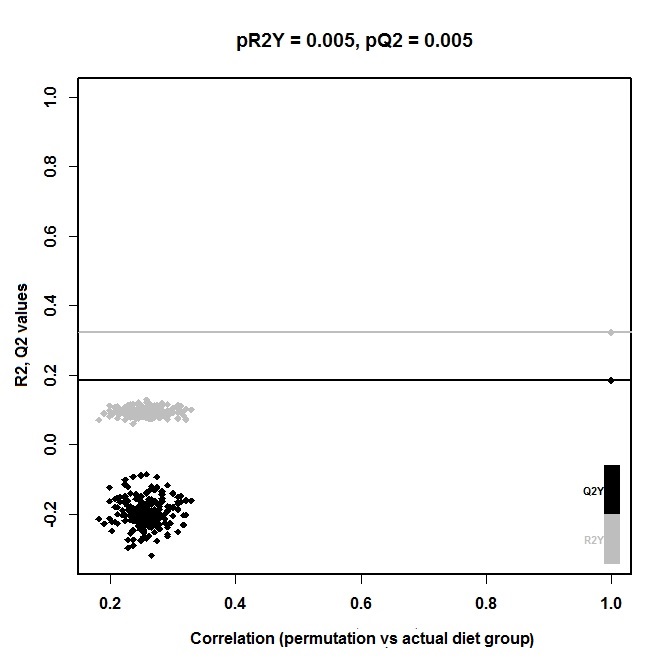
**

Figure S3. Validation of the partial least squares model of 207 NMR metabolite measures in four diet groups.

The plot shows the goodness of fit (R2Y) and goodness of prediction (Q2Y) for the actual model (diamonds with lines) and 200 permutation tests (diamonds without lines), in which diet group was assigned by random. Higher goodness of fit and prediction for the actual model than the permutation models, and negative values for goodness of prediction of the permutation models indicate no overfitting and thereby that the actual model is valid (Simeone *et al.* PLoS One 2014;9(7):e103030).

4

**
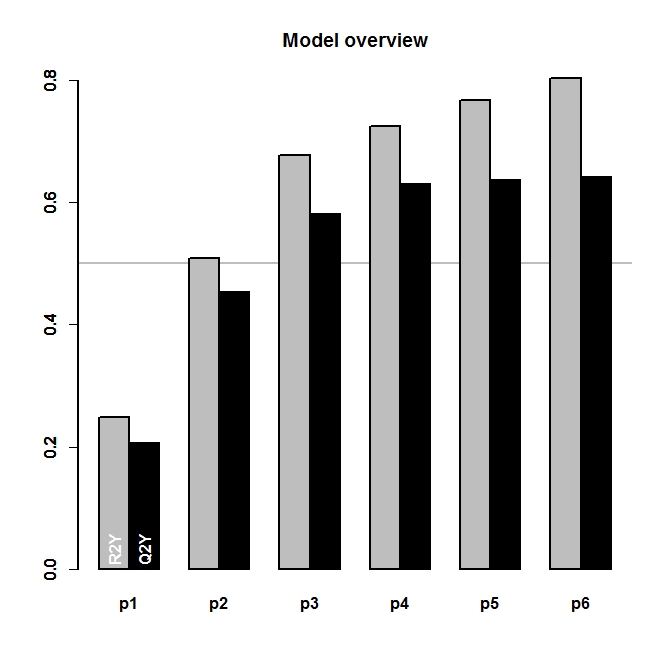
**

Figure S4. Cumulative goodness of fit (R2Y) and goodness of prediction (Q2Y) by the retained components (p1-p6) for the partial least squares model of 207 NMR metabolite measures in meat-eaters and vegans.

Seven-fold cross validation was used. R2Y = 0.803 and Q2Y = 0.642.

**5**

**
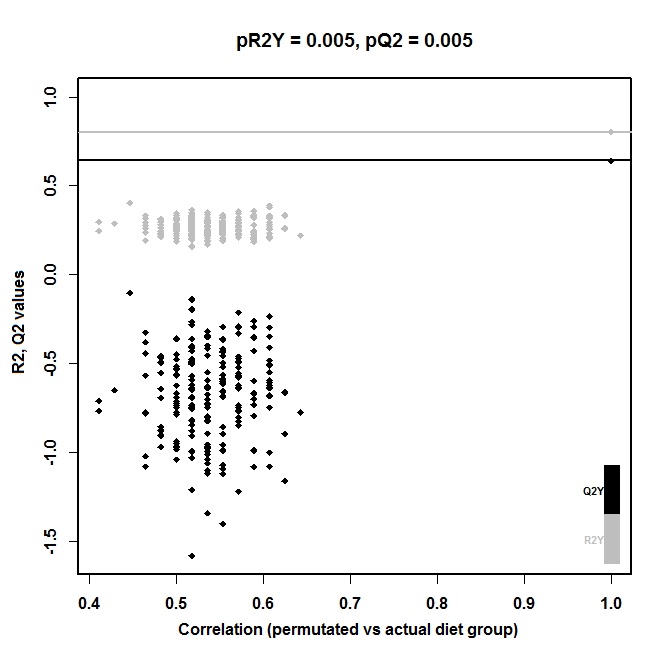
**

Figure S5. Validation of the partial least squares model of 207 NMR metabolite measures in meat-eaters and vegans.

The plot shows the goodness of fit (R2Y) and goodness of prediction (Q2Y) for the actual model (diamonds with lines) and 200 permutation tests (diamonds without lines), in which diet group was assigned by random. Higher goodness of fit and prediction for the actual model than the permutation models, and negative values for goodness of prediction of the permutation models indicate no overfitting and thus that the actual model is valid (Simeone *et al.* PLoS One 2014;9(7):e103030).

**6**

| **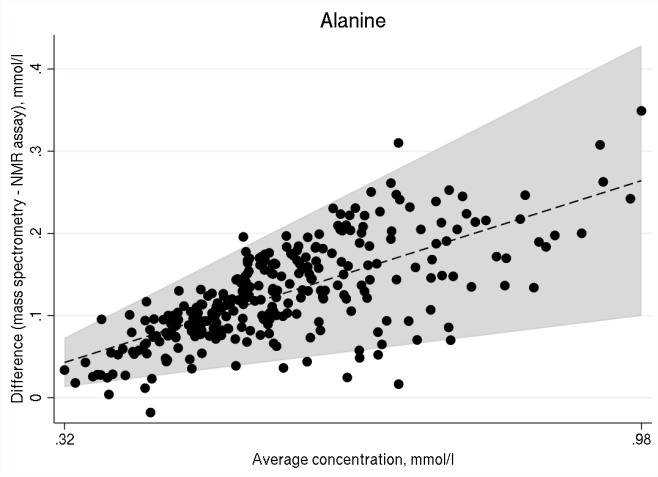** | **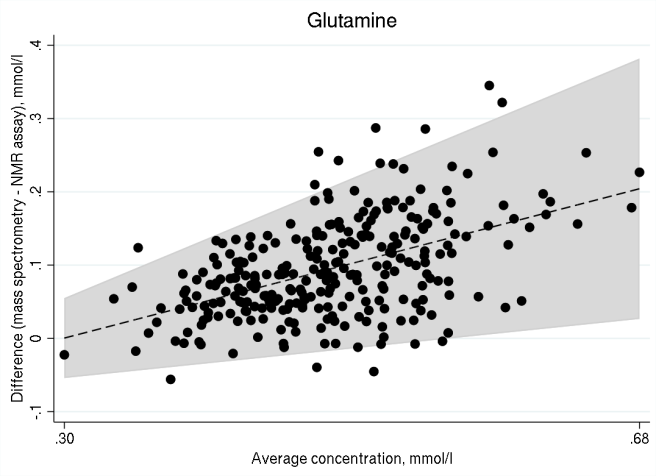** |
| --- | --- |
| **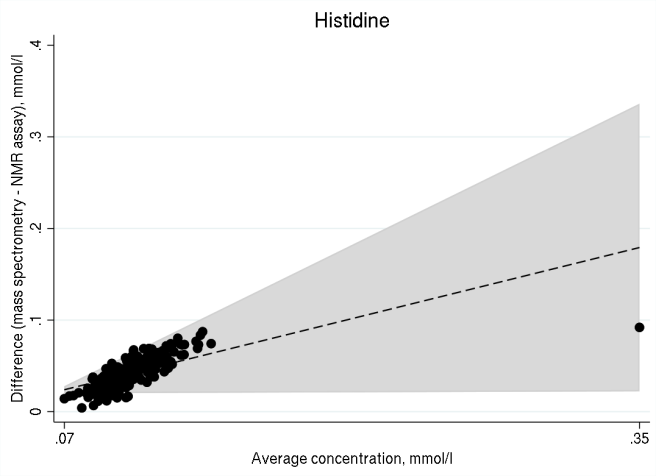** | **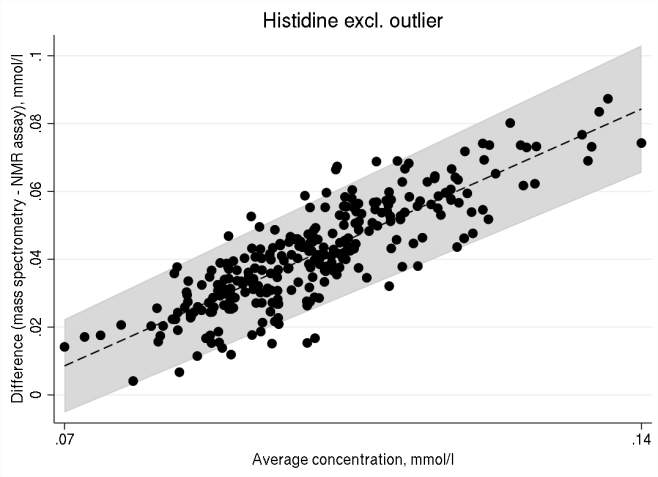** |
| **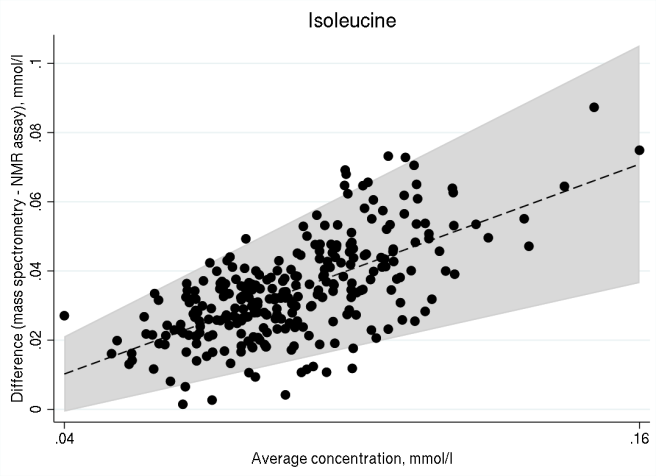** | **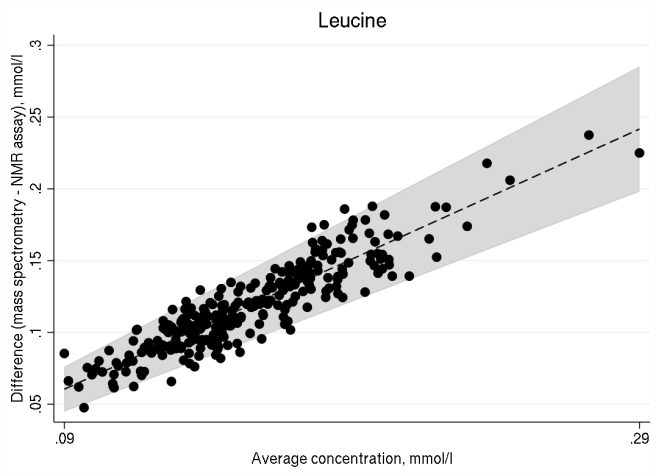** |
| **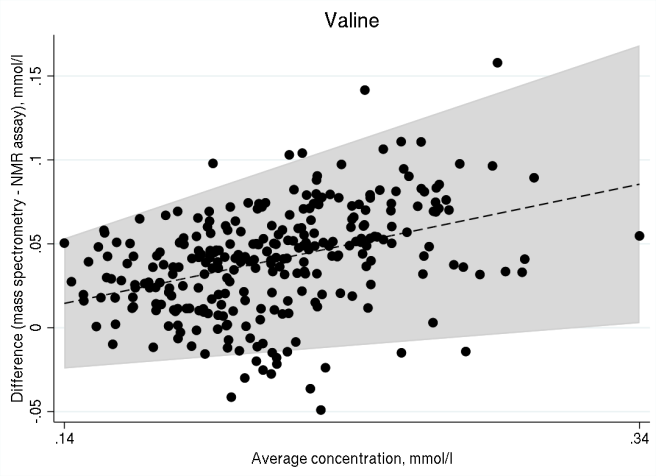** | **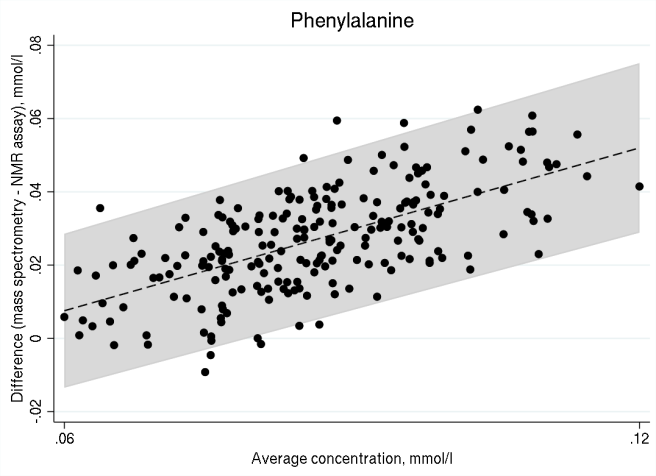**  7 |
| **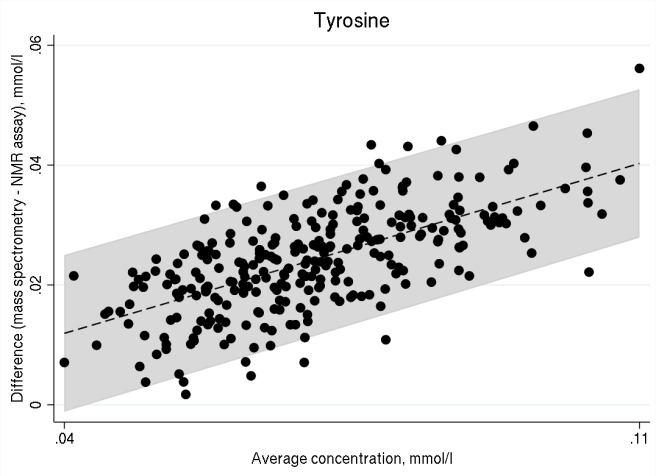** | **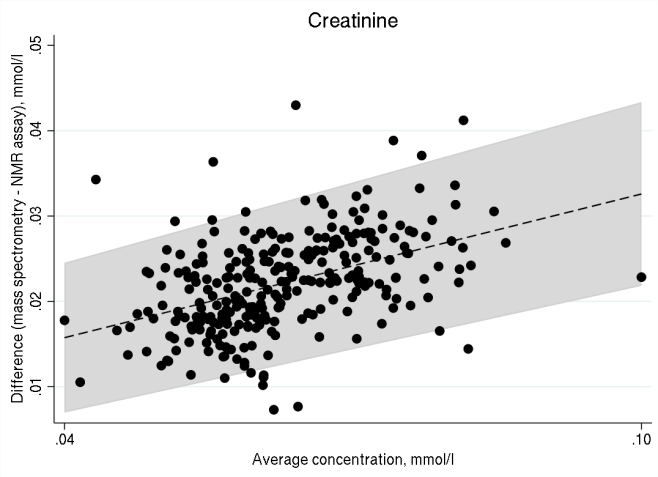** |
| **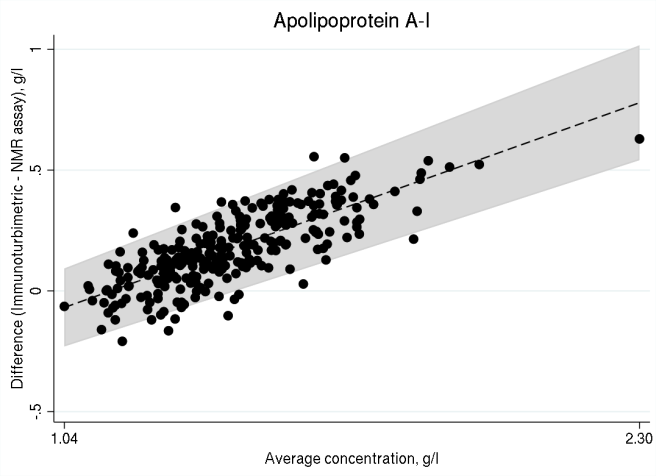** | **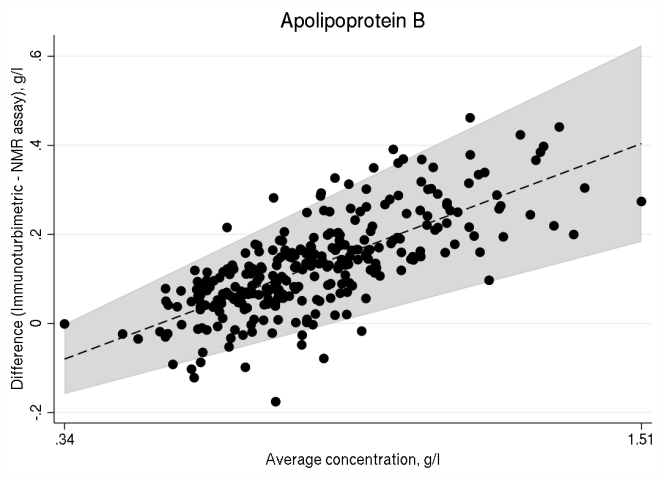** |
| **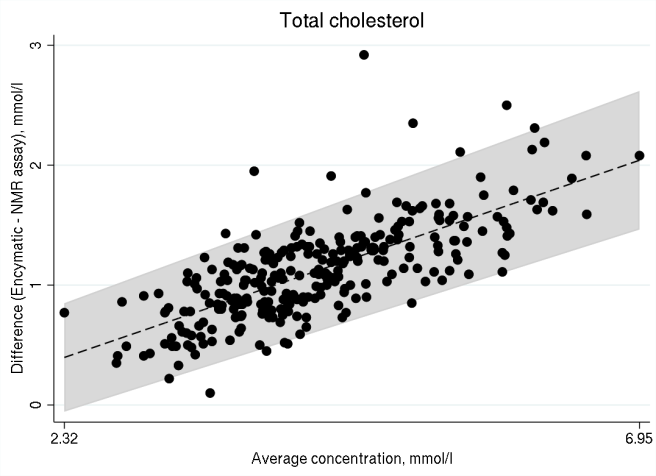** | **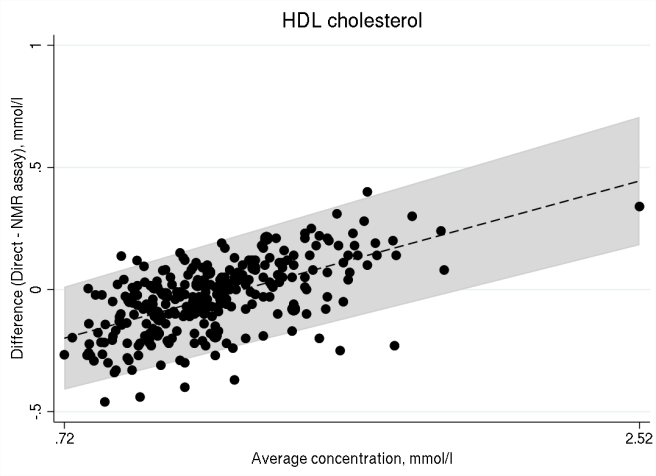** |
| **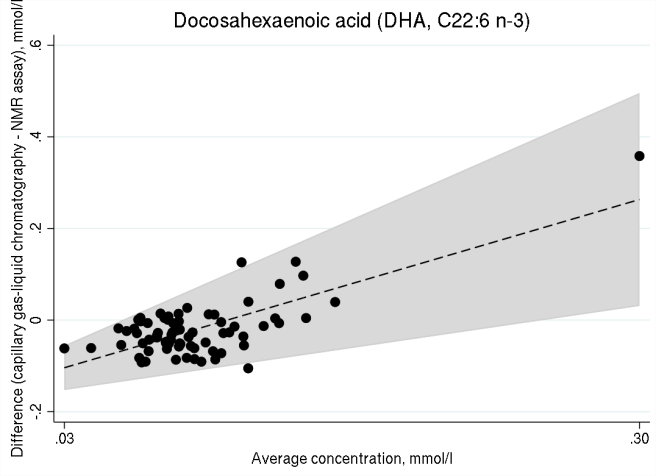** | **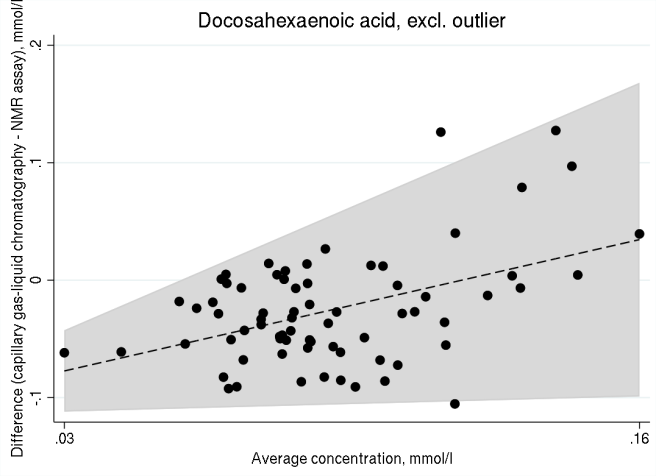**  8 |
| **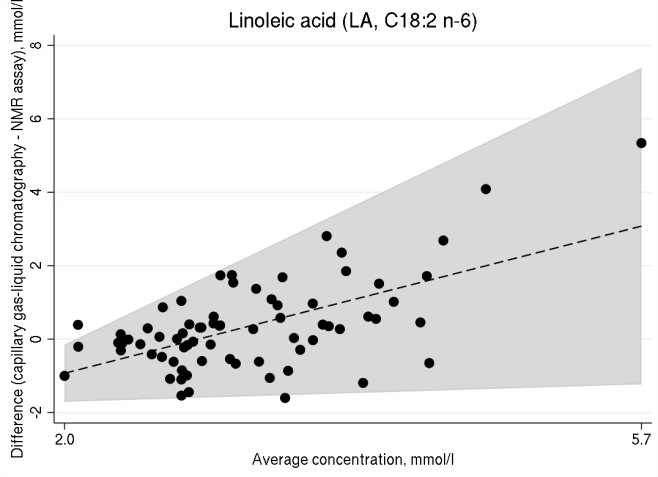** |  |

Figure S6. Bland−Altman plots comparing NMR metabolite concentrations to those measured using another method

Plasma amino acid and creatinine concentrations were measured using the mass spectrometry based Absolute*IDQ*^®^ p180 Kit (Biocrates Life Sciences AG, Innsbruck, Austria; Schmidt JA et al. Am J Clin Nutr 2015;102(6):1518-26). In serum, apolipoproteins were measured using an immunoturbimetric assay, total cholesterol was measured using an enzymatic assay and HDL cholesterol was measured directly (Bradbury KE et al. Eur J Clin Nutr 2014;68(2):178-83). Total plasma esterified and nonesterified fatty acids were measured using capillary gas-liquid chromatography (Rosell MS et al. Am J Clin Nutr 2005;82:327-34). More details are shown in Table S6.

9
